# Use of albumin as a risk factor for hospital mortality among burn patients in Brazil: non-concurrent cohort study

**DOI:** 10.1590/S1516-31802010000500009

**Published:** 2010-09-02

**Authors:** Gilson Caleman, José Fausto de Morais, Maria Eduarda dos Santos Puga, Rachel Riera, Álvaro Nagib Atallah

**Affiliations:** I MD, MSc, PhD. Full professor, Faculdade de Medicina de Marília (Famema), Marília, São Paulo, Brazil.; II MD, MSc, PhD. Full professor, Universidade Federal de Uberlândia (UFU), Uberlândia, Minas Gerais, Brazil.; III MSc. Research assistant, Brazilian Cochrane Center, São Paulo, Brazil.; IV MD, MSc. Attending physician in the Department of Emergency and Evidence-Based Medicine, Universidade Federal de São Paulo (Unifesp), and research assistant in the Brazilian Cochrane Center, São Paulo, Brazil.; V MD, PhD. Full professor in the Department of Emergency and Evidence-Based Medicine, Universidade Federal de São Paulo (Unifesp), and Director of the Brazilian Cochrane Center, São Paulo, Brazil.

**Keywords:** Serum albumin, Burns, Mortality, Inpatients, Cohort studies, Albumina sérica, Queimaduras, Mortalidade, Pacientes internados, Estudos de coortes

## Abstract

**CONTEXT AND OBJECTIVE:**

Among burn patients, it is common to use colloidal substances under the justification that it is necessary to correct the oncotic pressure of the plasma, thereby reducing the edema in the burnt area and the hypotension. The aim here was to assess the risk of hospital mortality, comparing the use of albumin and crystalloid solutions for these patients.

**DESIGN AND SETTING:**

Non-concurrent historical cohort study at Faculdade de Medicina de Marília; within the Postgraduate program on Internal and Therapeutic Medicine, Universidade Federal de São Paulo; and at the Brazilian Cochrane Center.

**METHODS:**

Burn patients hospitalized between 2000 and 2001, with registration in the Hospital Information System, who received albumin, were compared with those who received other types of volume replacement. The primary outcome was the hospital mortality rate. The data were collected from files within the Datasus software.

**RESULTS:**

39,684 patients were included: 24,116 patients with moderate burns and 15,566 patients with major burns. Among the men treated with albumin, the odds ratio for the risk of death was 20.58 (95% confidence interval, CI: 11.28-37.54) for moderate burns and 6.24 (CI 5.22-7.45) for major burns. Among the women, this risk was 40.97 for moderate burns (CI 21.71-77.30) and 7.35 for major burns (CI 5.99-9.01). The strength of the association between the use of albumin and the risk of death was maintained for the other characteristics studied, with statistical significance.

**CONCLUSION:**

The use of albumin among patients with moderate and major burns was associated with considerably increased mortality.

## RESUMO

**CONTEXTO E OBJETIVO:**

Em pacientes queimados é comum o uso de substâncias coloidais sob justificativa de que é necessário corrigir a pressão oncótica do plasma, reduzindo o edema na área queimada e a hipotensão. O objetivo foi avaliar o risco de mortalidade hospitalar, comparando o uso de albumina e soluções cristaloides para esses pacientes.

**TIPO DE ESTUDO E LOCAL:**

Estudo coorte histórico não concorrente na Faculdade de Medicina de Marília, no Programa de Pós-Graduação em Medicina Interna e Terapêutica da Universidade Federal de São Paulo e no Centro Cochrane do Brasil.

**MÉTODOS:**

Pacientes queimados hospitalizados entre 2000 e 2001, registrados no Sistema de Informações Hospitalares e que receberam albumina foram comparados com aqueles que receberam outros tipos de reposição volêmica. O desfecho primário foi a taxa de mortalidade hospitalar. Os dados foram coletados dos arquivos do programa Datasus.

**RESULTADOS:**

Foram incluídos 39.684 pacientes: sendo 24.116 pacientes com queimaduras moderadas e 15.566 pacientes com queimaduras graves. Entre os homens tratados com albumina, o *odds ratio* para o risco de morte foi 20,58 (intervalo de confiança IC 95% 11,28-37,54) para queimaduras moderadas e 6,24 (IC 5,22-7,45) para queimaduras graves. Entre as mulheres, esse risco foi de 40,97 para queimaduras moderadas (IC 21,71-77,30) e 7,35 para queimaduras graves (IC 5,99-9,01). A força da associação entre o uso de albumina e o risco de morte foi mantida para as outras características estudadas, com significância estatística.

**CONCLUSÃO:**

O uso de albumina entre pacientes com queimaduras moderadas e graves foi associado a aumento considerável da mortalidade.

## INTRODUCTION

Burns are defined as injuries to organic tissue resulting from trauma of thermal origin. The main consequences from such injuries are hemodynamic instability, shock, pain, hemorrhage, anemia, immunodepression and functional sequelae.^1,2^ The worldwide mortality rate due to severe burns is around 8% and the patients that die are mostly those presenting second and third-degree burns that affect more than 25% and 10% of the body surface, respectively, or when the burns affect the face or perineum, or cause severe respiratory injuries.^1,2^

The recommended treatment for these patients takes into account the principal intercurrences that are possible during hospitalization. Analgesia and fluid replacement using isotonic and hypertonic solutions have long been recommended and accepted.^2-4^

However, it is very common practice to use colloidal substances, and among these, albumin, under the justification that it is necessary to correct the oncotic pressure of the plasma, thereby reducing the edema in the burnt area and the possible hypotension.

The use of colloidal substances has been seriously questioned with regard to their efficiency, efficacy, effectiveness and safety. Recent systematic reviews and editorials have shown that the use of colloidal substances may cause increased hospital mortality among burn patients.^5-7^

In Brazil in the years 2000 and 2001, the National Health System (Sistema Único de Saúde, SUS) funded a mean of 12,300,000 hospitalizations per year, at a mean annual cost of five billion reais. The number of deaths was around 300,000, with a mortality rate of 2.7% (Tables 1 and 2). Burn patients were included among the surgical-clinical procedures and represented 0.91% of the total number of hospitalizations for this specialty, over these two years.

**Table 1.** Patient distribution according to the burn category, gender, age group, use of albumin and occurrence of deaths (Brazil 2000-2001)

Variables		Death in moderate burns				P	Death in major burns				P
		Yes		No			Yes		No		
		n	%	n	%		n	%	n	%	
**Gender**										
Male	77	60.63	13,946	58.13	0.569	739	54.82	8,800	61.99	< 0.001
Female	50	39.37	10,045	41.87		609	45.18	5,418	38.01	
**Total**		**127**	**100.00**	**23,991**		**100.00**	**1,348**	**100.00**	**14,218**		**100.00**
**Age group**										
≤ 19 years	20	15.75	14,925	62.28	< 0.001	323	54.91	7,801	54.91	< 0.001
20 to 39	23	18.11	5,085	21.22		415	26.71	3,795	26.71	
40 to 59	33	25.98	2,841	11.86		346	13.61	1,933	13.61	
≥ 60	51	40.16	1,115	4.64		264	4.78	679	4.78	
**Total**		**127**	**100.00**	**23,966** *		**100.00**	**1,348**	**100.00**	**14,208** †		**100.00**
**Mean length of hospital stay**										
≤ 7 days	73	57.48	17,290	72.1	< 0.001	944	70.03	5,557	39.08	0.009
8 to 14	26	20.47	4,687	19.5		234	17.36	5,190	36.50	
15 to 21	16	12.60	1,145	4.8		76	5.64	1,700	11.96	
≥ 22	12	9.45	869	3.6		94	6.97	1,771	12.46	
**Total**		**127**	**100.00**	**23,991**		**100.00**	**1,348**	**100.00**	**14,218**		**100.00**
**State where patient lived**										
São Paulo	21	16.54	2,807	11.70	< 0.001	247	18.32	2,215	15.58	< 0.001
Rio de Janeiro	17	13.39	1,019	4.25		138	10.24	713	5.01	
Minas Gerais	18	14.17	3,275	13.65		188	13.95	1,457	10.25	
Others	71	55.91	16,890	70.45		775	57.49	9,833	69.16	
**Total**		**127**	**100.00**	**23,991**		**100.00**	**1,348**	**100.00**	**14,218**		**100.00**
**Albumin**										
Used	29	22.83	253	1.05	< 0.001	419	31.08	901	6.34	< 0.001
Not used	98	77.17	23,738	98.95		929	68.92	13,317	93.66	
**Total**		**127**	**100.00**	**23,991**		**100.00**	**1,348**	**100.00**	**14,218**		**100.00**

* 27 patients with unknown age

† 10 patients with unknown age.

**Table 2.** Patient distribution and odds ratio estimates for the risk of death, according to burn category, use of albumin, deaths and sex. Brazil, 2000-2001 (P < 0.001)

		Moderate burns						Major burns					
		Deaths						Deaths					
Males	**Albumin**	**Yes**	**No**	**Total**	**%**	**OR**	**95% CI**	**Yes**	**No**	**Total**	**%**	**OR**	**95% CI**
	Used	14	149	163	8.59	20.58	(11.28 -.37.53)	225	577	802	28.05	6.24	(5.22 – 7.45)
	Not used	63	13,797	13,860	0.45			514	8,223	8,737	5.88		
	Total	77	13,946	14,023	0.55			739	8,800	9,539	7.75		
Females	**Albumin**	**Yes**	**No**	**Total**	**%**	**OR**	**95% CI**	**Yes**	**No**	**Total**	**%**	**OR**	**95% CI**
	Used	15	104	119	12.60	40.97	(21.71 – 77.30)	194	324	518	37.45	7.35	(5.99 – 9.01)
	Not used	35	9,941	9,976	0.35			415	5,094	5,509	7.53		
	Total	50	10,045	10,095	0.50			609	5,418	6,027	0.10		
	**Total**	**127**	**23,991**	**24,118**	**0.53**			**1,348**	**14,218**	**15,566**	**8.66**		

The ordinances of the Ministry of Health relating to severity of burn cases give the following definitions:

- Minor burn: Patients are considered to present minor burns if they have first and second-degree burns affecting up to 10% of their body area.
- Moderate burns: Patients are considered to present moderate burns if they have:
  First and second-degree burns affecting between 10% and 25% of their body area, or
  Third-degree burns affecting up to 10% of their body area, or Burns affecting their hands and feet.
- Major burns: Patients are considered to present major burns if they have:
  First and second-degree burns affecting 26% or more of their body area, or
  Third-degree burns affecting more than 10% of their body area, or Burns affecting the perineum.^3,4^

Patients are classified as presenting major burns if they were the victims of burns of any extent that were associated with one or more of the following situations: inhalatory injuries, polytrauma, cranial trauma, electrical trauma, shock, kidney failure, heart failure, liver failure, hemostasis disorders, pulmonary embolism, acute myocardial infarct, severe infectious conditions whether resulting from the burns or not, compartment syndrome and consumptive diseases.^3,4^

## OBJECTIVE

The aim of this study was to assess the risk of hospital mortality, comparing treatments using albumin and crystalloid solutions for patients with moderate and major burns.

## METHODS

This was a non-concurrent historical cohort study conducted at the Brazilian Cochrane Center, Universidade Federal de São Paulo (Unifesp) and Faculdade de Medicina de Marília (Famema).

This study included patients with moderate and major burns who were hospitalized at reference centers in 2000 and 2001 and who appeared in the records of the Hospital Information System of the Department of Information Technology, Ministry of Health (Datasus). Patients classified as presenting minor burns, or those for whom it was not possible to specify whether the registered procedure related to moderate or major burns, were excluded.

This was a convenience sample consisting of the total number of hospitalizations due to burns (49,343 cases in 2000 and 2001) that appear in the Hospital Information System, minus exclusions due to lack of definition of the procedures implemented and minus the cases of hospitalization due to minor burns.

The database used to collect the data was the Hospital Information System, which is stored in the Ministry of Health’s Datasus system. All the information in the database was grouped in relation to Authorizations for Hospitalization, i.e. as information relating to individuals. Authorizations for Hospitalization not only have a billing role within the national public SUS but also, above all, are a powerful instrument for epidemiological information. They contain data such as gender, date of birth, profession, type of hospitalization, International Classification of Diseases (ICD) coding, procedures undertaken, special procedures, number of days of hospitalization, reason for discharge or transfer, death, use of orthoses/prostheses, hemotherapy and attendance location.

The use of albumin is classified as a special procedure, and therefore it is possible to identify, on a case-by-case basis, the quantity used and the amount spent on the treatment. When albumin is not used, it is assumed that the patient received crystalloid solutions.

Statistical analysis was performed on the 39,684 burn cases (moderate and major burns) that were hospitalized through SUS in 2000 and 2001. The patients’ identities were kept confidential, in accordance with legal determinations.

The outcome measured was the death rate, during hospitalization, among patients with moderate and major burns, who were registered through SUS in 2000 and 2001.

The information groupings and analysis were performed by means of the Tab Win software. The chi-square test was used for analyzing associations between the variables and odds ratios (ORs) were calculated for occurrences of death among cohorts corresponding to patients who received albumin and patients who did not receive albumin, for groupings defined by the variables of sex, burn category, age group and mean length of hospital stay. To analyze combined associations between variables, binary logistic regression analysis was used, taking P < 0.05 to indicate significance for such associations. For ORs, 95% confidence intervals were constructed, and these were used in assessing the statistical significance of the results.

## RESULTS

The study included 39,684 patients. The sample represented all the patients hospitalized and registered through SUS in 2000 and 2001 with moderate and major burns.

The group with moderate burns consisted of 24,118 patients (60.77%). Among these, 282 (1.17%) received albumin during hospitalization and 29 died, thus representing a mortality rate of 10.28%. The vast majority of the patients with moderate burns (23,836; 98.83%) did not receive albumin. The mortality rate among this group was 0.41%. The total number of deaths in this group was 127 patients, with a mortality rate of 0.53%.

The group with major burns consisted of 15,566 patients (39.23%), of whom 1,320 received albumin. Among these, there were 419 deaths, representing a mortality rate of 31.74%. The other 14,245 patients did not receive albumin during their hospitalization, and the mortality rate among these patients was 6.51%. The total number of deaths among patients with major burns was 1348, representing a mortality rate of 8.66%.

### Results according to variables

Table 1 presents the distributions of patients with moderate and major burns in relation to gender, age group, length of hospital stay, federal state where the patient was hospitalized, use of albumin and the main outcome (death). Examination of the data showed that there was a statistically non-significant association between sex and death due to moderate burns (P = 0.569) and a statistically significant association between sex and death due to major burns (P < 0.0001). The percentages of deaths among males due to moderate and major burns were 60.63% and 54.82%, respectively, and thus were greater than the percentages for females. The percentages of deaths among patients with moderate burns were 0.55% for males and 0.50% for females. Among the patients with major burns, the percentages were 7.75% and 10.10%, respectively. It was observed that the mortality rate among patients with major burns was greater for males. Among the patients with moderate burns, the mortality rates were similar for females and males.

Comparison between the age groups and death due to moderate burns (P < 0.001) and between the age groups and death due to major burns (P < 0.001) showed that there was a statistically significant difference between the age distributions for deaths due to moderate and major burns (P < 0.001). The older the age group was, the greater the risk of death was, both for the group with moderate burns and for the group with major burns.

Other statistically significant associations were found between the mean length of stay in hospital and death due to moderate burns (P < 0.001) and between the mean length of hospital stay and death due to major burns (P < 0.001). These showed that there was a statistically significant difference in the distributions of mean length of hospital stay, in relation to deaths due to moderate and major burns (P < 0.001). This indicated that the mortality rate was greater when the mean length of hospital stay was shorter, i.e. ≤ 7 days versus 8 to 14 days.

In relation to the federal state where the patient lived and death due to moderate burns (P = 0.001), a statistically significant association was observed. There was also a statistically significant association between federal state and death due to major burns (P < 0.008). However, there was no statistically significant difference in the distribution of federal states where the patients lived, between the deaths among patients with moderate burns and the deaths among patients with major burns (P = 0.471). A high proportion of the deaths in the group of patients with moderate burns occurred among the patients living in the State of São Paulo.

Another observation was that there was a statistically significant association between the use of albumin and death among the patients with moderate and major burns (P < 0.001). This showed a statistically significant difference in the distribution of the use of albumin between the deaths due to moderate burns and the deaths due to major burns (P < 0.001). There was a high proportion of deaths among the patients with moderate burns who received albumin.

### Specific results

The data in Table 2 show that, for all the groups defined by gender, burn category and use of albumin, there was a greater risk of death in the group that received albumin than in the group that did not receive it. Specifically, among the male patients with moderate burns, the risk of death in the group that received albumin was around 20 times greater; among the male patients with major burns, this risk was around six times greater; among the female patients with moderate burns, this risk was around 40 times greater; and among the female patients with major burns, this risk was around seven times greater. Analysis of the results found indicated that they were statistically significant, with P < 0.001, and showed that the use of albumin was associated with increased hospital mortality.

For all the groups defined by age group, burn category and use of albumin, there was a greater risk of death in the group that received albumin than in the group that did not receive albumin. Among the patients with moderate burns who were less than 19 years old, the risk of death in the group that received albumin was around 13 times greater than in the group that did not receive albumin; among the patients with major burns who were 19 years of age or under, this risk was around seven times greater; among the patients with moderate burns who were in the age groups from 20 to 39 years, 40 to 59 years and 60 years and over, these risks were 40, 29 and 12 times greater, respectively; and among the patients with major burns, the risks for these groups were six, six and four times greater, respectively (Table 3).

**Table 3.** Patient distribution and odds ratio estimates for the risk of death, according to burn category, use of albumin, deaths and age ranges. Brazil, 2000-2001 (P < 0.001)

		Moderate burns						Major burns					
		Deaths						Deaths					
≤ 19 years	**Albumin**	**Yes**	**No**	**Total**	**%**	**OR**	**95% CI**	**Yes**	**No**	**Total**	**%**	**OR**	**95% CI**
	Used	2	109	111	1.80	13.59	(3.14 – 58.86)	95	427	522	18.20	7.20	(5.56 – 9.32)
	Not used	20	14,816	14,836	0.13			228	7,374	7,602	2.94		
	Total	22	14,925	14,947	0.15			323	7,801	8,124	3.90		
20 – 39 years	**Albumin**	**Yes**	**No**	**Total**	**%**	**OR**	**95% CI**	**Yes**	**No**	**Total**	**%**	**OR**	**95% CI**
	Used	9	79	88	10.23	40.57	(17.06 – 96.49)	150	292	442	33.93		
	Not used	14	5,006	5,020	0.28			264	3,503	3,767	7.00		
	Total	23	5,085	5,108	0.48			414	3,795	4,209	9.84		
40 – 59 years	**Albumin**	**Yes**	**No**	**Total**	**%**	**OR**	**95% CI**	**Yes**	**No**	**Total**	**%**	**OR**	**95% CI**
	Used	10	42	52	19.23	28.08	(12.99 – 64.65)	114	134	248	45.97	6.60	(4.96 – 8.77)
	Not used	23	2,799	2,822	0.82			232	1,709	1,941	11.42		
	Total	33	2,841	2,874	1.14			346	1,843	2,189	15.18		
≥ 60 years	**Albumin**	**Yes**	**No**	**Total**	**%**	**OR**	**95% CI**	**Yes**	**No**	**Total**	**%**	**OR**	**95% CI**
	Used	8	22	30	26.67	12.15	(5.41 – 27.31)	60	48	108	55.56	3.87	(2.56 – 5.83)
	Not used	41	1,093	1,134	3.62			204	631	835	24.43		
	Total	49	1,115	1,164	4.21			264	679	943	28.00		
	**Total**	**127**	**23,966** *	**24,903**	**0.53**			**1,347^†^**	**14,118** ‡	**15,555**	**8.66**		

* 25 patients with unknown age

† 1 patient with unknown age

‡ 10 patients with unknown age

OR = odds ratio; CI = confidence interval.

For all the groups defined by the mean length of hospital stay, burn category and use of albumin, there was a greater risk of death in the group that received albumin than in the group that did not receive albumin. Among the patients with moderate burns who stayed in hospital for less than seven days, the risk of death in the group that received albumin was around 73 times greater; among the patients with major burns who stayed in hospital for less than seven days, this risk was around 12 times greater; among the patients with moderate burns who stayed in hospital for 8 to 14 days, 15 to 21 days and more than 21 days, these risks were 30, 10 and 5 times greater, respectively; and among the patients with major burns, the risks for these groups were 16, 11 and 5 times greater, respectively (Table 4).

**Table 4.** Patient distribution and odds ratio estimates for the risk of death, according to burn category, use of albumin, deaths and mean length of hospital stay. Brazil, 2000-2001 (P < 0.001)

		Moderate burns						Major burns					
		Deaths						Deaths					
≤ 7 days	**Albumin**	**Yes**	**No**	**Total**	**%**	**OR**	**95% CI**	**Yes**	**No**	**Total**	**%**	**OR**	**95% CI**
	Used	11	39	50	22.00	78.48	(38.43 – 160.28)	216	134	350	61.71	12.01	(9.55 – 15.10)
	Not used	62	17,251	17,313	0.36			728	5,423	6,151	11.84		
	Total	73	17,290	17,363	0.42			944	5,557	6,501	14.52		
8 – 14 days	**Albumin**	**Yes**	**No**	**Total**	**%**	**OR**	**95% CI**	**Yes**	**No**	**Total**	**%**	**OR**	**95% CI**
	Used	8	66	74	10.81	30.19	(12.69 – 71.81)	106	249	355	29.86	16.43	(12.33 – 21.90)
	Not used	18	4,619	4,637	0.39			128	4,941	5,069	2.53		
	Total	26	4,685	4,711	0.55			234	5,190	5,424	4.31		
15 – 21 days	**Albumin**	**Yes**	**No**	**Total**	**%**	**OR**	**95% CI**	**Yes**	**No**	**Total**	**%**	**OR**	**95% CI**
	Used	6	64	70	8.57	10.13	(3.57 – 28.76)	44	193	237	18.57	10.74	(6.65 – 17.34)
	Not used	10	1,081	1,091	0.92			32	1,507	1,539	2.01		
	Total	16	1,145	1,161	1.38			76	1,700	1,776	4.27		
≥ 22 days	**Albumin**	**Yes**	**No**	**Total**	**%**	**OR**	**95% CI**	**Yes**	**No**	**Total**	**%**	**OR**	**95% CI**
	Used	4	82	86	4.65	4.80	(1.41 – 16.28)	53	48	101	52.47	5.7	(3.76 – 8.80)
	Not used	8	787	795	1.01			41	631	672	6.10		
	Total	12	869	881	1.36			94	679	773	12.16		
	**Total**	**127**	**23,989**	**24,116**	**1.36**			**1,348**	**13,126**	**14,474**	**8.66**		

OR = odds ratio; CI = confidence interval.

The data in Table 5 also show that, for all the groups defined by the federal state where the patient lived, burn category and use of albumin, there was a greater risk of death in the group that received albumin than in the group that did not receive albumin. Specifically, among the patients with moderate burns who lived in the State of São Paulo, the risk of death in the group that received albumin was 51 times greater; among the patients with major burns who lived in the State of São Paulo, this risk was around seven times greater; among the patients with moderate burns who lived in the States of Rio de Janeiro, Minas Gerais and other states, these risks were 114, 13 and 30 times greater, respectively; and among the patients with major burns who lived in these locations, the risks for these groups were three, ten and six times greater, respectively.

**Table 5.** Patient distribution and odds ratio estimates for the risk of death, according to burn category, use of albumin, deaths and location (federal state). Brazil, 2000-2001 (P < 0.001)

		Moderate burns						Major burns					
		Deaths						Deaths					
São Paulo	**Albumin**	**Yes**	**No**	**Total**	**%**	**OR**	**95% CI**	**Yes**	**No**	**Total**	**%**	**OR**	**95% CI**
	Used	11	63	74	14.66	47.91	(19.63 – 116.91)	114	249	363	31.40	6.77	(5.10 – 8.98)
	Not used	10	2,744	2,754	0.33			133	1,966	2,099	6.34		
	Total	21	2,807	2,828	0.71			247	2,215	2,462	10.03		
Rio de Janeiro	**Albumin**	**Yes**	**No**	**Total**	**%**	**OR**	**95% CI**	**Yes**	**No**	**Total**	**%**	**OR**	**95% CI**
	Used	6	68	74	8.11	7.63	(2.74 – 21.25)	58	147	205	28.29	2.90	(1.08 – 4.25)
	Not used	11	951	962	1.14			79	567	646	12.23		
	Total	17	1,019	1,036	1.64			137	714	851	16.10		
Minas Gerais	**Albumin**	**Yes**	**No**	**Total**	**%**	**OR**	**95% CI**	**Yes**	**No**	**Total**	**%**	**OR**	**95% CI**
	Used	2	31	33	6.66	13.01	(2.87 – 59.00)	100	150	250	40.00	9.90	(7.10 – 13.81)
	Not used	16	3,226	3,242	0.52			88	1,307	1,395	6.31		
	Total	18	3,257	3,275	0.58			188	1,457	1,645	7.69		
Others	**Albumin**	**Yes**	**No**	**Total**	**%**	**OR**	**95% CI**	**Yes**	**No**	**Total**	**%**	**OR**	**95% CI**
	Used	10	91	101	9.90	30.60	(15.20 – 61.63)	146	356	502	41.01	6.23	(5.05 – 7.68)
	Not used	61	16,811	16,872	0.38			630	9,477	10,107	6.23		
	Total	71	16,902	16,973	0.42			776	9,833	10,609	7.69		
	**Total**	**127**	**23,985**	**24,112**	**0.53**			**1,348**	**14,219**	**15,567**	**8.66**		

OR = odds ratio, CI = confidence interval.

### Analysis of combined associations by means of logistic regression

Logistic regression analysis established the values for the variables of age group, mean length of hospital stay and federal state where the patient was hospitalized. The aim was to study the risk of death among the patients who received albumin when the combined effect of the other variables was taken into consideration. It was shown that the risk of death (expressed by ORs) among the patients who received albumin was OR = 7.39 (95% CI: 6.44 to 8.48) for the established variable of age group; OR = 12.06 (95% CI: 10.37 to 14.04) for the established variable of mean length of hospital stay; and OR = 8.56 (95% CI: 7.51 to 9.76) for the established variable of federal state where the patient was hospitalized. Although the P-value in the Hosmer and Lemeshow test was less than 0.05 and the Nagelkerke R-square was greater than 0.10, the P-value of the test model (which was less than 0.05) and the AUROC (area under the receiver operating characteristic curve) of 0.12 indicated that the model had satisfactory discriminatory power.

## DISCUSSION

The analyses on the results from this study showed that there was a very high association between the use of human albumin and hospital mortality, for any presentation of patients with burns and in relation to the different variables analyzed (sex, age, location of hospitalization and length of hospital stay). The mortality rate increased with the use of albumin among patients with moderate and major burns.

Among the patients with moderate burns (less severe), for all the characteristics studied, the use of albumin was a risk factor for increased hospital mortality. Among the female patients, the risk of death was 40 times greater (95% CI: 21.71-77.30) in the group treated with albumin, in relation to the control group. Among the male patients, the risk was 20 times greater (95% CI: 11.28-37.54). Although less intensely, this relationship was repeated for all the other characteristics studied, and the association was confirmed by the logistic regression method.

Among the patients with major burns (more severe), the risk of death through the use of albumin was also significant. The risk of death was seven times greater (95% CI: 5.99-9.01) among the female patients and six times greater (95% CI: 5.22-7.45) among the male patients. Just like among the patients with moderate burns, this relationship was maintained with the other variables.

The explanation that the increased mortality rate may have been influenced by the severity and extent of the burns does not stand up to examination, because the risk of death was higher among the patients with moderate burns, i.e. among those less severely affected. Some strong points can be highlighted in this study, among which is the fact that the sample was large and representative, considering that it included all the patients with moderate and major burns (39,684 patients) who were hospitalized and registered through SUS in the years 2000 and 2001. The exploratory analysis was extensive and the strength and consistency of the association were confirmed by the statistical tests. The independence of the analysis was sustained by information appearing in the Ministry of Health’s Datasus database, i.e. the information is in the public domain and can be accessed for any type of rechecking.

The results from the present study confirm the evidence brought to light in the various studies carried out by the Cochrane Injuries Group Albumin Review (systematic review with meta-analysis).^6,7^ Retrospective studies may contain failings relating to the trustworthiness of the information. In the case of the present study, such failings have been overcome, because there are some studies that compare the information appearing in the SUS Hospital Information System (authorizations for hospitalization) with the medical records, with the aim of analyzing the pertinence of the Datasus information and validating it.^8,9^ Following an analysis of 1036 authorization forms for hospitalization registered with a main diagnosis of acute myocardial infarct in the municipality of Rio de Janeiro in 1997, the results obtained demonstrated that the quality of the diagnosis was satisfactory, with a high percentage confirmation of 91.7% (95% CI: 78.3-94.2).

Some studies have tried to show the opposite, i.e. concluding in favor of using albumin, given that no harm was shown through its use.^10,11^ However, the information appearing in these studies does not allow such a conclusion. In burn cases, as already seen through the present study, the use of albumin cannot be recommended.

The multicenter Safe clinical trial published in 2004 compared the effect of using albumin with saline solution on the mortality rate among a heterogeneous population of 6997 patients hospitalized in intensive care units in 16 tertiary-level hospitals in Australia and New Zealand between November 2000 and June 2003.^12^ It showed that the use of albumin did not enable any change in the mortality rate, in comparison with the use of isotonic solutions to achieve the expected benefit in relation to mortality, thereby reaffirming the evidence from previous reviews.

The present study, which involved a non-concurrent cohort, corroborates and consolidates the evidence found by the Cochrane reviews in relation to burn patients, i.e. that the use of albumin is a risk factor for hospital mortality among such patients. Among the patients with moderate and major burns who were treated with albumin, the risk of death was significantly higher in comparison with the risk run by patients who did not undergo this treatment.

Another possibility would be that only the patients with the severest burns should receive albumin. However, the observed increase in mortality was greater among the less severely affected patients (moderate burns). In other clinical situations, the Cochrane studies objectively indicated that the use of albumin did not produce any benefit for patients, in comparison with the use of crystalloids.^6,7^

Another important matter relates to the possible problems associated with administering albumin in clinical practice, which include the following:

– Products derived from blood are not pure and there are large quality differences in albumin preparations. The presence of endotoxins, polymers, bradykinin and other proteins may alter the quality of the albumin and result in anaphylaxis.^13^
– A large proportion of critically ill patients present increased capillary fragility and extravasation of albumin to interstitial areas, which may cause pulmonary edema. The pulmonary lymphatic system only has limited capacity for removing large volumes of fluids from interstitial areas, thereby giving rise to greater vulnerability towards edema. There is evidence to suggest that pulmonary dysfunction among critically ill patients does not depend on the osmotic pressure of the plasma. The deleterious effects of albumin on the lungs may be produced by various factors, including secondary hypervolemia due to antinatriuresis and antidiuresis, and by changes in secondary oncotic pressure due to increased extravascular albumin in the lungs. Burn patients treated with colloids may have a high level of water retention in the lungs for up to seven days after receiving the injury.^13^
– There may be worsening of the inflammatory processes among patients treated with albumin, because this affects the hemodynamics of the microcirculation. Another effect, paradoxically, is a decrease in the glomerular filtration rate, through a mechanism that is still unknown.^13^ A likely explanation is that the albumin increases the oncotic pressure inside the peritubular vessels, thus causing a decrease in the excretion of sodium and water. Albumin, contrary to what is believed, does not boost the natriuresis of furosemide.^13^ It is more likely that it aggregates furosemide in the renal tubules, thereby promoting its inactivation and diminishing its diuretic effect.^13^
– Several other effects have been reported, such as the occurrence of urticaria, fever and shivers.^13^

With regard to the efficacy and effectiveness of the use of albumin, the literature available and the results from the present study have indicated that there is no evidence to support continuing to use it indiscriminately. If these arguments are not enough, comparison of the efficiency of treatments using albumin and crystalloid solutions shows that the differences are significant. The price difference between one product and the other is of the order of up to 100-fold. The amount paid by hospitals in Brazil for 20% albumin solution ranges from R$ 37.00 to R$ 155.00, while the SUS price tables reimburse R$ 55.00 for a 50-ml flask. The annual mean number of flasks paid for by SUS over the last seven years (2000 to 2006) was 247,230, with an annual mean expenditure of R$ 13,597,650.00, for all clinical conditions. The total expenditure on albumin in Brazil can be expected to greatly exceed this amount, if the expenditure by the supplementary health system and direct expenditure by society are taken into account, but unfortunately these data are not available. On the other hand, the market price for units of crystalloid solutions ranges from R$ 0.77 to R$ 2.55.

The results presented have indicated that, among burn patients, the use of albumin are deleterious, thus confirming the investigations carried out by the Injuries Group Albumin Review.^6,7^

There is still much controversy in relation to whether or not to use albumin in healthcare services. Nonetheless, most of the studies that have been published defend the need to carry out further research on this subject.

## CONCLUSIONS

The use of albumin for patients who are classified as presenting moderate and major burns is associated with a great increase in mortality. Therefore, until it is proven to the contrary, human albumin must not be used any more for burn patients at any level, and its use must be restricted to clinical research and physiopathological studies.
